# *N*-Acyl Homoserine Lactone Production by the Marine Isolate, *Dasania marina*

**DOI:** 10.3390/microorganisms12071496

**Published:** 2024-07-22

**Authors:** Fnu Alimiran, Samuel David, Scott Birks, Athenia Oldham, Douglas Henderson

**Affiliations:** 1 Department of Biology, University of Texas of the Permian Basin, Odessa, TX 79762, USA; almira199625@gmail.com (F.A.); oldham_a@utpb.edu (A.O.); 2 Department of Chemistry, Southern Oregon University, Ashland, OR 97520, USA; davids1@sou.edu (S.D.);

**Keywords:** *Dasania*, *N*-acyl homoserine lactone production, biofilm

## Abstract

*Dasania marina* (isolate SD1D, with 98.5% sequence similarity to *Dasania marina* DMS 21967 KOPRI 20902) is a marine bacterium that was isolated from ballast tank fluids as part of a biofilm study in 2014. Our previous work indicated that although this strain produced no detectable biofilm, it was the only isolate to produce *N*-acyl homoserine lactones (AHLs) in assays using the broad-range reporter strain, *Agrobacterium tumefaciens* KYC55. The goal of the current study was to determine the types of AHL molecules produced by the *D. marina* isolate using gas chromatography–mass spectroscopy (GCMS) and C4- to C14-AHL as standards. A time course assay indicated that the *D. marina* strain produced the highest level of AHLs at 20 h of growth. When extracts were subjected to GCMS, detectable levels of C8- and C10-AHL and higher levels of C12-AHL were observed. Interestingly, several biofilm-forming isolates obtained from the same source also produced detectable amounts of several AHLs. Of the isolates tested, a strain designated SD5, with 99.83% sequence similarity to *Alteromonas tagae* BCRC 17571, produced unstable biofilms, yet detectable levels of C6-, C8-, C10- and C12-AHL, and isolate SD8, an *Alteromonas oceani S35* strain (98.85% sequence similarity), produced robust and stable biofilms accompanied by detectable levels of C8- and C12-AHL. All isolates tested produced C12-AHL at higher levels than the other AHLs. Results from this study suggest that quorum sensing and biofilm formation are uncoupled in *D. marina*. Whether the suite of AHLs produced by this isolate could modulate biofilm formation in other strains requires further study.

## 1. Introduction

Quorum sensing (QS) is the cell-to-cell communication system that bacteria use to couple cellular density to group behaviors such as biofilm formation, sporulation, or bioluminescence [1,2]. QS involves the synthesis, secretion, and detection of chemical signaling molecules called autoinducers, and the bacterial cells’ physiological response(s) to those signals. Different groups of bacteria respond to different autoinducers. Gram-positive bacteria synthesize and respond to autoinducing peptides (AIPs), Gram-negative bacteria synthesize and respond to *N*-acyl homoserine lactones (AHLs), and both groups respond to autoinducer-2 (AI-2), providing support for both intra- and interspecies communication [3,4]. While recent QS studies have focused on identifying compounds to disrupt one or more of these QS pathways in microorganisms that causes disease [3,5,6,7], there is still much to learn in other fields, especially maritime, where strategies to disrupt communication in microorganisms to mitigate the accumulation of biofilm on ships (i.e., ballast tanks and hulls) could significantly decrease maintenance costs and material damage [8,9].

Bacteria have the ability to attach to and form a biofilm (communities of microorganisms encased in protective polymer substances) on most surfaces [10]. The formation of these protective structures is controlled directly by QS [1]. Previously, we showed that a Gram-negative marine isolate identified as *Dasania marina* produced detectable levels of AHLs using a broad-range reporter strain, *A. tumefaciens KY5CC*, but produced no observable biofilm [11]. In this report, we sought to confirm the result and expand our studies to identify the types of AHLs produced by this strain using gas chromatography–mass spectrometry (GCMS). Our results revealed that AHL production for *D. marina* peaked at approximately 20 h and it predominantly synthesized C12-AHL, with measurable but lower levels of C8- and C10-AHL, indicating a decoupling of QS from biofilm formation in this strain. To our knowledge, this is the first report of AHL in the strain and only the second publication since its identification in 2007 [12].

## 2. Materials and Methods

**Bacterial strains and growth conditions**. The marine bacteria used in the study were obtained from the ballast tanks of 4 US Navy ships [11]. The marine strains were *Dasania marina* (SD1D), *Alteromonas oceani S35* strain (SD8), and *Alteromonas tagae* (SD5). The marine bacteria stocks were maintained in Difco Marine Broth 2216 (MB) (Becton, Dickinson and Company, Sparks, MD, USA) with 30% glycerol at −80 °C. The strains were grown for 2 d on MB agar at 26 °C, and a single colony was transferred to 4 mL of MB broth and grown for 2 d at the same temperature at 100 rpm. For the time course assays with *D. marina* SD1D and AHL extractions that were analyzed by GCMS, 100 μL of the 2 d broth culture was inoculated into 100 mL of MB broth, grown at 26 °C, and samples were removed and analyzed at the indicated times.

**AHL detection.** A modified version of the procedure by Joelsson [13] was used to recover AHLs from the supernatant of bacterial cultures. Briefly, 4 mL of cultures grown in marine broth (MB) were centrifuged at 13,500× *g* for 3 min and the supernatant was subjected to extraction with 4 mL of ethyl acetate. The residue was resuspended in 400 μL acetonitrile (10× extract) and stored in a glass vial at −20 °C. A variation of the method by Cha [14] was used to detect AHLs on solid media using the AHL-reporter strain *A. tumefaciens* KYC55/pJZ372, pJZ384, and pJZ410 [15]. The plasmids in the reporter strain serve the following functions: pJZ372 contains the *traI-lacZ* fusion, pJZ384 contains *traR* under the control of the T7 promoter, and pJZ410 contains the T7 RNA Pol gene. TraR is the activator which is responsive to a number of different AHLs. *traI* is an AHL biosynthesis gene which is controlled by TraR, so that when TraR binds an extraneously added AHL, it will lead to the expression of the *traI-lacZ* fusion detected when X-gal or ONPG is present and broken down by β-galactosidase. The reporter strain was grown on Luria agar with antibiotics at 26 °C for 2 d, and isolated colonies were inoculated into 0.6 mL AT medium with antibiotics and grown for 24 h. The cells were seeded into 20 mL of warm melted AT agar (0.8%) supplemented with antibiotics and 40 μL of 20 mg/mL X-gal and poured to make AT agar plates. Five μL aliquots from the 10× extract were spotted onto AT agar plates, and plates were incubated at 26 °C for 24 h. To quantify AHL in broth cultures, β-galactosidase assays were conducted as described by Joelsson [13].

**AHL Analysis by Gas Chromatography–Mass Spectrometry.** The following standards were purchased from Caymen Chemicals (Ann Arbor, MI, USA): *N*-butyryl-l-Homoserine Lactone (C4-AHL, Cat#10007898), *N*-hexanoyl-l-Homoserine Lactone (C6-HL, Cat#10011197), *N*-octonyl-l-Homoserine Lactone (C8-AHL, Cat#10011199), *N*-decanoyl-Homoserine Lactone (C10-AHL, Cat#10011201), *N*-dodecanoyl-Homoserine Lactone (C12-AHL, Cat#10011201), and *N*-tetradecanoyl-Homoserine Lactone (C14-AHL, Cat#10011200). Method optimization was performed using 5.0 ppm ethanolic AHL standards analyzed using a GCMS-TQ8040 gas chromatograph–triple quadrupole mass spectrometer (Shimadzu Scientific Instruments, Columbia, MD, USA). The injector was maintained at 200 °C and 1.0 μL of the sample was injected with a split ratio of 10.0. Separations were achieved using an RTX-5ms capillary column (30 m, 0.25 mm ID, 0.25 μm film thickness, Restek, Bellefonte, MD, USA) using a He carrier gas at a constant flow of 0.80 mL/min. The column temperature was initially 150 °C, increased to 275 °C at 15 °C/min and held for 6.00 min. Analytes were ionized by chemical ionization using methane as a reagent gas (flow rate 0.8 mL/min). Following the collection of full scans for each standard, the multiple reaction monitoring (MRM) conditions were established using the Shimadzu Smart MRM program version 4.45 for each of the analytes and can be found in Appendix A, Table A1. For each AHL, a quantification and qualification transition were monitored, and identification was based on retention time and ion ratios. The transfer line temperature was 280 °C, and the ion source was maintained at 230 °C. For quantification of the six most common HLSs (C4-HL, C6-HL, C8-HL, C10-HL, C12-HL and C-14HL), AHL standards at concentrations of 0.1, 1, 10, 20, 50, and 100 ppm were used to create calibration curves showing acceptable linearity over this range (R^2^ > 0.97). Peaks for C4 and C14 were not well resolved at any concentration and were thus omitted from standard curve analysis. It is also worth noting that C6 and C12 peaks were not resolved at 0.1 ppm, so this data point was not included in calibration curves. Limits of detection and limits of quantification were determined using linear regression statistics of standard curves as follows: LOD = 3.3 σ/m and LOQ = 10 σ/m; σ is the standard deviation of the response of the curve and m is the slope. Bacterial extracts were analyzed using the optimized MRM method; compound identification was based on the retention time, and both quantification and qualification transitions.

## 3. Results

**Figure 1:**
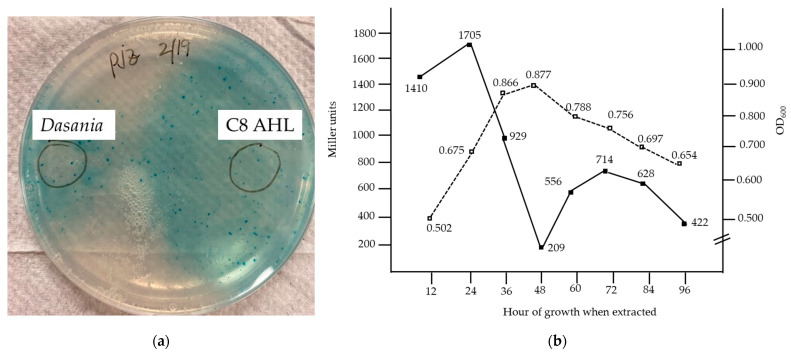
AHL production by *D. marina*. (**a**) AHL production of extracts from *D. marina* spotted on AT medium seeded with X-gal and AHL reporter strain *Agrobacterium tumefaciens* KYC55/pJZ372, pJZ384, and pJZ410. (**b**) Time course of AHL production as assessed by β-galactosidase assays of reporter strain mixed with extracts from *D. marina*. Miller units (left vertical axis) are indicated with a solid square and solid line and the growth (OD600) (right vertical axis) is indicated by dotted line and open squares.

***D. marina* produces AHL.** A plate assay was performed in which the reporter strain *A. tumefaciens* KYC55/pJZ372, pJZ384, pJZ410 was seeded into AT media containing X-gal, and extracts from *D. marina* were spotted on the plate. This reporter strain responds to a wide range of exogenous AHL including C4- to C18-HL and 3-oxo-C4- to 3-oxo-C16-HL. The extracts from *D. marina* produced a turquoise zone on the plate comparable to synthetic C8-AHL that was spotted on the plate indicative of AHL production (Figure 1a). A time course assay was performed on extracts from *D. marina* grown in marine broth to determine at what time point *D. marina* produced the most AHL. The strain was grown over a 96 h period and every 12 h extracts were obtained and used to perform β-galactosidase using the reporter strain. As indicated in Figure 1b, which shows a representative time course, the highest number of Miller units occurred at 24 h, indicating the highest AHL production at that time point. A Miller unit is a standard measure of β-galactosidase activity from the *lacZ* gene. This peak was seen 12 h before the maximum growth level occurred. A smaller peak was also observed at 72 h, 14 h after the OD_600_ declined from its peak of 0.877. AHL production from this second peak may be associated with the expression of metabolic genes required later in growth. AHL production in this second peak was not further characterized. To pinpoint the time when the most AHL was generated (large peak), additional time course assays were carried out in which samples were taken every 4 h between 12 and 28 h. The results of these assays indicated that the highest number of Miller units occurred at 20 h. This indicated that the analysis of AHL production should be carried out on the strain grown for 20 h.

**Figure 2:**
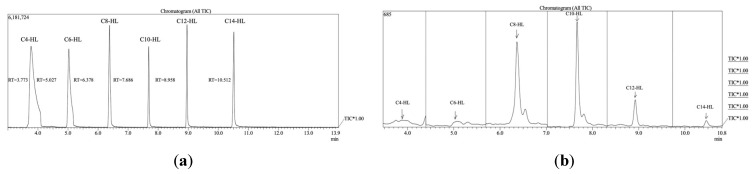
Chromatographs from GCMS analyses of (**a**) AHL standards (at 5 ppm) used in the study and (**b**) *D. marina* extracts.

***D. marina SD1D, A. tagae* SD5, *A. oceani* SD8 produce several different AHLs.** Extracts from *D. marina* and the other isolates were analyzed using GCMS and compared to the six standards used (C4-, C6-, C8-, C10-, C12-, and C14-AHL) (Figure 2a). Chromatograms showed elevated levels of C8, C10 and C12 AHLs in *D. marina* (Figure 2b). Quantification of the peaks indicated that C12 was the most abundant AHL produced (Table 1).

**Figure A1:**
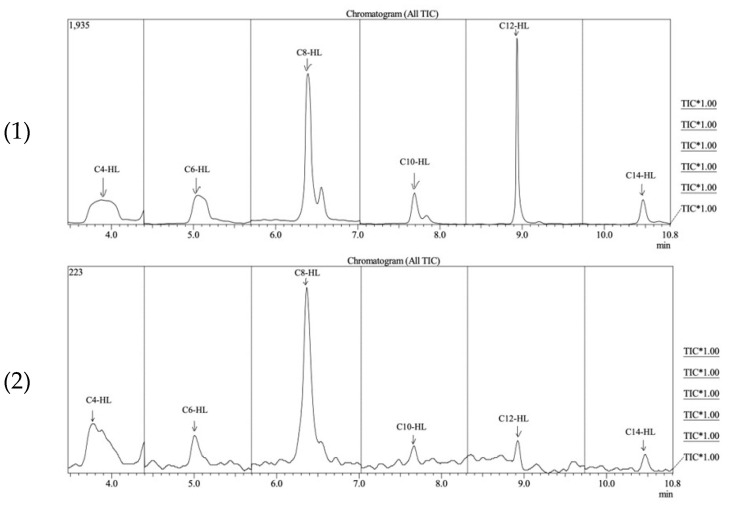
(**1**) *A. tagae* SD1 and (**2**) *A. oceani*.

Chromatographs were also obtained for *A. tagae* SD5 and *A. oceani* SD8 (Appendix A; Figure A1 panel 1 and 2, respectively). Quantification of the peaks indicated that *A. tagae* produced C6-, C8-, C10- and C12-AHL, whereas *A. oceani* produced only C8- and C12-AHL at detectable levels. In both of these strains, like *D. marina*, C12-AHL was the most abundant. A quantification analysis was not performed or included for C4- and C14-AHL due to the lack of calibration data.

## 4. Discussion

The investigation into *D. marina* unveils a paradoxical scenario where the robust production of AHLs coexist with an undetectable biofilm formation, suggesting alternative functions of AHLs beyond biofilm development in this strain. In the time course assay with *D. marina*, two peaks of AHL production were observed, one earlier in growth when the OD_600_ was increasing and one when the OD_600_ was in decline. We analyzed the types of AHL produced only in the first larger peak. The second peak of AHL production may have been associated with starvation stress observed with *Pseudomonas aeruginosa*, as referred to by Lee and Zhang (2015) in their review on the hierarchy of quorum sensing networks in that organism [16].

Further, comparisons with biofilm-forming strains from the same ballast tank source, *Alteromonas tagae* (isolate SD5) and *Alteromonas oceani* (isolate SD8) underscore the diversity in QS mechanisms among marine bacteria. Despite all three strains predominantly producing C12-AHL, their biofilm formation characteristics vary significantly [11], indicating the complex interplay between QS regulation and bacterial behavior across species.

The prevalence of C12-AHL in the three strains raises intriguing questions about its potential significance for bacterial survival and adaptation. A recent review by Sahreen et al. featured a range of AHLs involved in biofilm formation, and interestingly, three of the studies summarized therein associated C12-AHL with quorum quenching (inhibiting biofilm formation) [4]. Future investigations could explore the specific functions of C12-AHL and their interplay with other AHLs to unravel their broader implications for bacterial physiology and ecological interactions.

In a prior investigation led by Mari Conceição Aquino de Sá [17], a comparative proteomic analysis was conducted between strains of *Corynebacterium pseudotuberculosis* isolated from goats, distinguishing between those capable of forming biofilms (CAP J4) and those that could not. The non-biofilm-forming strain (CAP3W) exhibited a deficiency in certain proteins associated with biofilm formation and exopolysaccharide biosynthesis. This suggests that other factors such as specific proteins and environmental conditions may play pivotal roles in biofilm development. Future research endeavors could delve into proteomic analyses to explore differences in the expression of specific proteins, thereby shedding more light on the intricacies of biofilm formation.

## Figures and Tables

**Table 1:** Quantification of AHLs produced by *D. marina* isolate SD1D, *A. tagae* isolate SD5, and *A. oceani* isolate SD8 ^1^.

Organism	C6	C8	C10	C12
*D. marina* (isolate SD1D)	UD	4.46	5.12	8.72
*A. tagae* (isolate SD5)	5.94	4.50	5.09	8.82
*A. oceani* (isolate SD8)	UD	4.43	UD	8.70

^1^ In parts per million (ppm); UD = undetectable.

## Data Availability

The original contributions presented in the study are included in the article/Appendix A, further inquiries can be directed to the corresponding author.

## Appendix A

**Table A1:** MRM method parameters for AHL detection and quantification.

Analyte	RT (min)	MRM Start Time (min)	MRM End Time (min)	Quantification		Qualification	
				MRM Transition	Collision Energy	MRM Transition	Collision Energy
*N*-butyryl-l-Homoserine Lactone (C4-AHL)	3.77	3.48	4.73	172.00 > 71.10	11	172.00 > 102.10	6
*N*-hexanoyl-l-Homoserine Lactone (C6-AHL)	5.03	4.73	5.71	200.00 > 102.10	4	200.00 > 99.10	7
*N*-otonyl-l-Homoserine Lactone (C8-AHL)	6.38	5.71	7.05	228.00 > 57.10	24	228.00 > 127.10	6
*N*-decanoyl-Homoserine Lactone (C10-AHL)	7.69	7.05	8.34	256.00 > 102.10	7	256.00 > 155.20	6
*N*-dodecanoyl-Homoserine Lactone (C12-AHL)	8.96	8.34	9.27	284.00 > 102.10	6	284.00 > 57.10	23
*N*-tetradecanoyl-Homoserine Lactone (C14-AHL)	10.51	9.27	10.80	312.00 > 102.10	6	312.00 > 57.10	23

**Table A2:** Limits of detection and quantification for AHL standards.

Compound	LOD (ppm)	LOQ (ppm)
C4-AHL	UD	UD
C6-AHL	21.05	63.77
C8-AHL	20.95	63.48
C10-AHL	24.01	72.75
C12-AHL	29.21	88.52
C14-AHL	UD	UD

LOD = limit of detection; LOQ = limit of quantification.
